# Deglycosylation of eukaryotic-expressed flagellin restores adjuvanticity

**DOI:** 10.1038/s41541-023-00738-3

**Published:** 2023-09-26

**Authors:** Koemchhoy Khim, Sao Puth, Kamalakannan Radhakrishnan, Tien Duc Nguyen, Youn Suhk Lee, Che-Hun Jung, Shee Eun Lee, Joon Haeng Rhee

**Affiliations:** 1 Clinical Vaccine R&D Center, Chonnam National University, Hwasun-gun, Jeonnam Republic of Korea; 2 Combinatorial Tumor Immunotherapy MRC, Chonnam National University Medical School, Hwasun-gun, Jeonnam Republic of Korea; 3 Immunotherapy Innovation Center, Hwasun-gun, Jeonnam Republic of Korea; 4 Department of Microbiology, Chonnam National University Medical School, Hwasun-gun, Jeonnam Republic of Korea; 5 Department of Pharmacology and Dental Therapeutics, School of Dentistry, Chonnam National University, Gwangju, Republic of Korea; 6 Department of Chemistry, Chonnam National University, Gwangju, Republic of Korea

**Keywords:** Adjuvants, Adjuvants

## Abstract

Flagellin, the TLR5 agonist, shows potent adjuvant activities in diverse vaccines and immunotherapies. *Vibrio vulnificus* flagellin B expressed in eukaryotic cells (eFlaB) could not stimulate TLR5 signaling. Enzymatic deglycosylation restored eFlaB’s TLR5 stimulating functionality, suggesting that glycosylation interferes with eFlaB binding to TLR5. Site-directed mutagenesis of N-glycosylation residues restored TLR5 stimulation and adjuvanticity. Collectively, deglycosylated eFlaB may provide a built-in adjuvant platform for eukaryotic-expressed antigens and nucleic acid vaccines.

Flagellin, the structural component of bacterial flagella, comprises highly conserved D0 and D1 and hypervariable D2 and D3 domains. The D1 domain of flagellin is important for its immunomodulatory function as it contains the Toll-like receptor 5 (TLR5) binding domain [1–3]. Flagellin possesses potent adjuvant activity through activating innate immunity and modulating adaptive immune responses via the TLR5-nuclear factor kappa B (NF-κB) signaling [2, 4–6]. We have reported that bacterial flagellin, *Vibrio vulnificus* FlaB, serves as a versatile adjuvant that can be applied to a wide range of vaccines and immunotherapies [7–10].

Post-translation modifications modulate the biological activity of proteins. N- and/or O-glycosylation is important to maintain protein structure, biological activity, molecular stability, solubility, and reduced immunogenicity [11,12]. It has been reported that glycosylation of the SARS-CoV-2 spike protein makes it more virulent by enhancing the binding affinity to the ACE2 receptor and evading the immune responses. Recombinant SARS-CoV-2 spike antigens require proper glycosylation for inducing neutralizing antibodies [13,14]. We have shown that the genetic fusion of vaccine antigens with FlaB generates built-in adjuvanted vaccines that induce superior protective immune responses to mixture formulations [7]. Given that prokaryotic expression systems do not provide post-translational glycosylation, for example, a eukaryotic-expression system is required for built-in flagellin-adjuvanted vaccines composed of glycosylated antigens in SAR-CoV-2 spike proteins. In this regard, we first checked TLR5 stimulating activities of eukaryotic expressed FlaB (eFlaB), which should possibly be glycosylated in eukaryotic cells since bioinformatic analysis predicted several glycosylating amino acids.

To this end, the codon-optimized *flaB* gene was cloned into the eukaryotic expression vector pSectag2B, and transfected into Expi293 cells. His-tagged eFlaB was purified using nickel resin and analyzed by SDS-PAGE and immunoblotting (Fig. 1a). eFlaB exhibited increased molecular mass than the calculation based on amino acid contents (Fig. 1a). eFlaB showed significantly attenuated TLR5 stimulating activity in pilot studies. We hypothesized that post-translational glycosylation of eFlaB may have interfered with the TLR5 binding since we found putative N-glycosylating amino acid near the TLR5 binding domain. To prove this, we treated WT eFlaB with peptide N-glycosidase F (PNGase F) and observed that molecular mass decreased and TLR5-stimulating activity was restored (Fig. 1b, c). As for the TLR5 stimulating activities, eFlaB was significantly lower than prokaryotic-expressed FlaB (pFlaB) (eFlaB, EC_50_ 13.83 nM vs pFlaB, EC_50_ 3.32 nM). The PNGase F treatment recovered TLR5-stimulating activity (from EC_50_ 13.83 nM to 3.88 nM, Fig. 1c). The defective TLR5-stimulating activity of eFlaB could be attributed to N-glycosylation. We virtually predicted putative N-glycosylating residues in eFlaB using the NetNGlyc-1.0 software (https://services.healthtech.dtu.dk/service.php?NetNGlyc-1.0). Five putative glycosylating residues were predicted at N83, N101, N139, N273, and N330. Interestingly, three residues were located near the TLR5 binding domain (N83, N101, N139) (Supplementary Fig. 1b, c). We mutated each residue individually and in combinations (Fig. 2a). Corroborating deglycosylation results, the site-directed mutagenesis of N-glycosylating residues resulted in reduced molecular masses and restored TLR5-stimulating activity (Fig. 2b, c). Specifically, single mutation N83A and multiple mutations N83/101A, N83/101/139A, N83/101/139/273A, and N83/101/139/273/330A induced TLR5-mediated NF-κB activation. Adding N101A to N83A did not further restore TLR5 stimulating activity while adding N139A, N273A, and N330A to the double mutation (N83/101A) resulted in full recovery of TLR5 stimulating functionality comparable to pFlaB. We also confirmed the N-glycosylation of eFlaB by using the characteristic of ConA that specifically binds with N-glycosylated proteins (Supplementary Fig. 2). These results suggest that glycosylation in distant amino acid residues should have also inhibited the eFlaB-TLR5 interaction (Fig. 2c). Employing a co-immunoprecipitation (Co-IP) experiment, it was also confirmed that glycosylation interfered with the FlaB-TLR5 binding and deglycosylation resulted in higher affinity binding (Fig. 2d).

**Fig. 1:**
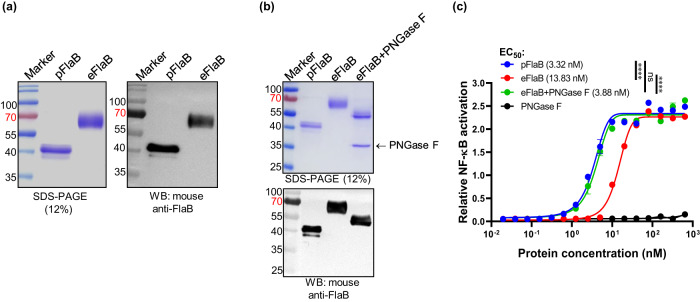
Characteristics of the recombinant proteins. **a** Characterization of eukaryotic-(eFlaB) or prokaryotic- (pFlaB) expressed *Vibrio vulnificus* FlaB by sodium dodecyl sulfate-polyacrylamide gel electrophoresis (SDS-PAGE) and western blot analysis (WB) with mouse anti-pFlaB serum. **b** Characterization of Peptide N-glycosidase F (PNGaseF)-digested deglycosylated eFlaB by SDS-PAGE and WB analysis. **c** Comparison of TLR5-dependent NF-κB stimulating activity of pFlaB, eFlaB, and PNGaseF-treated eFlaB. EC_50_ values were analyzed by a two-tailed Student’s *t*-test. ****P < 0.0001; ns non-significant.

**Fig. 2:**
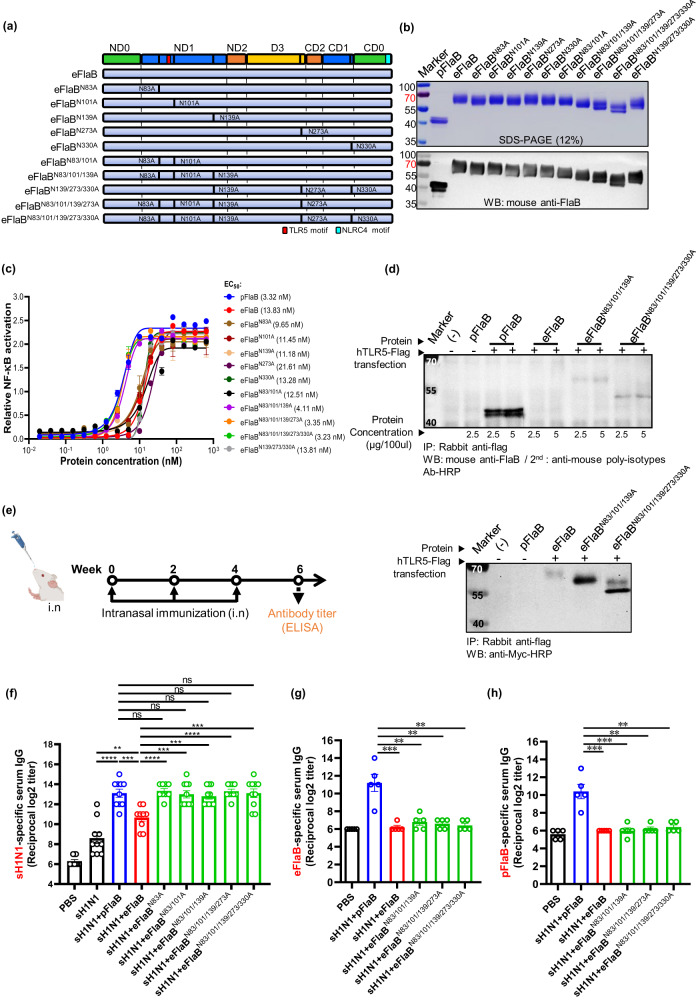
Deglycosylation of eukaryotic-expressed FlaB restores TLR5 stimulation and adjuvanticity. **a** Schematic view of predicted N-glycosylation sites of FlaB and mutated eFlaB variants. Five N-glycosylation sites were predicted in eFlaB using NetNGlyc-1.0 and N-glycosylation mutants were constructed by site-direct mutagenesis. **b** Characterization of FlaB variants by SDS-PAGE and WB with mouse anti-pFlaB. **c** Determination of TLR5-mediated NF-κB stimulating activity of FlaB variants. **d** Determination of TLR5 binding with FlaB variants by co-immunoprecipitation. **e** Immunization schedule. **f** determination of sH1N1-specific serum IgG levels by ELISA (*n* = 10/group). Determination of eFlaB- (**g**) and pFlaB- (**h**) specific serum IgG levels by ELISA. Data are presented as the mean ± SEM. All data were analyzed by a two-tailed Student’s *t*-test. **P < 0.01; ***P < 0.001; ****P < 0.0001; ns non-significant.

We then tested the adjuvant activity of the mutated variants in a murine influenza vaccination model. Briefly, BALB/c mice were intranasally immunized with split H1N1 (sH1N1) antigen alone or in combination with FlaB variants (Fig. 2e). Compared to the eFlaB-adjuvanted group, the N-glycosylation site mutation variants significantly potentiated sH1N1-specific antibody response as comparable level to pFlaB (Fig. 2f). Of note, eFlaB variants showed a similar level of in vivo adjuvanticity, while their in vitro TLR5 stimulating activities were less in N83A and N83/101A variants, suggesting possible deglycosylation in vivo. Interestingly, in contrast to pFlaB, eFlaB variants did not induce recognizable pFlaB- or eFlaB-specific antibodies (Fig. 2g, h). This result indicates that eFlaB variants do not stimulate B cell responses as de-immunized pFlaB did [15]. Eukaryotic post-translational modifications other than glycosylation may have made eFlaB hypoimmunogenic, which requires further clarification.

Here, we showed that FlaB manufactured in eukaryotic cells was glycosylated and defective in TLR5 binding (Fig. 2d), suggesting that glycans around the TLR5 binding domain interfered with FlaB interaction with FlaB-TLR5 interaction. Glycan removal either by deglycosylating enzyme or mutating putative N-glycosylation residues restored the TLR5 stimulating functionality. The present findings enable the use of mutated eFlaBs for the development of all-in-one flagellin-adjuvanted protein vaccines that require glycosylation to generate a protective immune response. The variant FlaB sequences could be used for DNA/mRNA vaccines designed to express all-in-one flagellin adjuvanted fusion antigens in vivo. Among putative N-glycosylation target residue, N83 appeared to play a dominant role in inhibiting FlaB from binding to TLR5 since the single mutation N83A could provide sufficient adjuvanticity of eFlaB in inducing sH1N1-specific antibody responses.

Moreover, multiple site-directed mutations on N-glycosylation residues (N83/101A, N83/101/139A, N83/101/139/273A, and N83/101/139/273/330A) also restored the adjuvanticity of eFlaB to the same level as single mutation N83A in a murine influenza vaccine model (Fig. 2f). However, multiple mutations without N83A (N139/273/330A) failed to restore eFlaB induced TLR5-activation (Fig. 2c), corroborating the hypothesis that glycosylation near the TLR5 binding domain interfered with the receptor-ligand interaction. We also found that eFlaB and its variants made discrete structures and remained stable even under room temperature as evidenced by SDS-PAGE and NF-κB reporter assay (Supplementary Fig. 3). Previously we showed that pFlaB could be de-immunized by deleting 19 amino acid B cell epitopes [15]. Here we show that eFlaB variants did not induce FlaB-specific antibody responses without deletion of the immunogenic epitope. Other post-translational modifications than N-glycosylation may have masked immunogenic B cell epitopes. Thus, eFlaB variants would be more suitable for vaccine and immunotherapeutic developments.

## Methods

### Ethics

All animal experimental procedures were approved by the Chonnam National University Institutional Animal Care and Use Committee under the protocol CNU IACUC-H-2021-51. Animal research facility maintenance and experimental procedures were conducted in rigorous accordance with the guidelines of the Animal Welfare Act legislated by the Korean Ministry of Agriculture, Food and Rural Affairs.

### Plasmid construction

The construction and production of FlaB from the prokaryotic expression system (pFlaB) were described previously [16]. Briefly, the *Vibrio vulnificus* flagellin B (*flaB*) gene was cloned into the pTYB12 vector. Then, the resulting expression plasmid was transformed into *E. coli* ER2566 (New England Biolabs, Beverly, MA) by electroporation. The bacteria were spread onto the LB plate containing ampicillin antibiotic. Then, we cultured the bacteria and added the inducer 0.5 mM IPTG (isopropyl-β-D-thiogalactopyranoside) at 0.6–0.8 OD600 of culture in 20 °C for 18–20 h. Next, we pelleted down the bacteria and resuspended in a lysis buffer (20 mM Tris-Cl [pH 7.5], 500 mM NaCl, 1 mM EDTA [pH 8.0], 0.1% Triton X-100, 0.1% Tween 20, 20 M phenylmethylsulfonyl fluoride) and sonicated (Vibra Cell VCX500; Sonics & Materials, Inc., Newtown, CT) on an ice bed. After centrifugation at 35,000 × *g* for 30 min, the supernatant was mixed with chitin resin bead and remove nonspecific protein by slowly dropped at 4 °C until finish the volume and washed with washing buffer (20 mM Tris-Cl [pH 7.5], 500 mM NaCl, 1 mM EDTA [pH 8.0]). Finally, the protein was eluted with the elution buffer (20 mM Tris-Cl [pH 7.5], 500 mM NaCl, 1 mM EDTA [pH 8.0], 50 mM 1,4-dithiothreitol) and confirmed the purity of pFlaB by SDS-PAGE. For eukaryotic expression FlaB (eFlaB), a codon-optimized *flaB* gene sequence was synthesized by Bioneer Inc., South Korea. The *flaB* gene was amplified by PCR reaction using forward primer, 5’-CCC**AAGCTT**GCCGTCAACGTGAACACCAA-3’ containing *HindIII* (**AAGCTT**) site and reverse primer, 5’-ATAGTTTA**GCGGCCGC**GCCCAGCAGGGAGAGGGCGC-3’ containing *NotI* (**GCGGCCGC**) site. The amplified *flaB* and eukaryotic expression vector pSectag2B (Invitrogen, V900-20) were digested by restriction enzymes *HindIII* and *NotI* at 37 °C overnight. Then, we ligated the *flaB* with the pSectag2B vector by T4 DNA ligase (enzynomics, M001S) at room temperature for 2 h. The ligated plasmid was transformed into *Escherichia coli* TOP10 competent cells and spread onto an LB agar plate containing ampicillin (Supplementary Table 1). The sequence was confirmed by the Macrogen online sequencing system (http://dna.macrogen.com/kor/).

### DNA transfection, protein purification, and characterization

The mammalian cell line Expi293^TM^ is obtained from Thermofisher Scientific. DNA transfection was done using ExpiFectamine following the manufacturer protocol (Thermofisher, A14635). Briefly, the Expi293^TM^ cells were cultured at 37 °C, 8% CO_2_, and 125 rpm shaking speed of incubator (eppendorf, S41I230011) till three or more passages before transfection. On the day of transfection (day 1), 3 × 10^6^ Expi293^TM^ cells/ml were seeded and transfected with purified pSectag2B plasmids containing the *flaB* gene. On day 2, enhancer I and enhancer II were added and cultured until day 4. On day 4, we transferred the culture medium to harvest bottles and centrifuged at 35,000 × *g* at 4 °C for 20 min. The supernatant was collected and mixed with 10x lysis buffer (500 mM NaH_2_PO_4_, 1.5 M NaCl, and 100 mM imidazole, pH = 8) in a ratio of 9:1. The mixture was added to the equilibrated Niken column containing 3 ml of Ni-NTA agarose bead (Qiagen, 30230), for 30 ml of the protein mixture, at 4 °C. To remove the non-specific proteins, the column was washed with washing buffer I (50 mM NaH_2_PO_4_, 300 mM NaCl, and 10 mM imidazole, pH=8) and washing buffer II (50 mM NaH_2_PO_4_, 300 mM NaCl, and 20 mM Imidazole, pH = 8). The protein was eluted with elution buffer (50 mM NaH_2_PO_4_, 300 mM NaCl and 250 mM imidazole, pH = 8) at room temperature for 15 min. The buffer was changed to phosphate-buffered saline (PBS) using centrifugal filter tubes (Merck, UFC801024). The recombinant eFlaB protein purity was confirmed by sodium dodecyl sulfate-polyacrylamide gel electrophoresis (SDS-PAGE) and subsequent Western blot analysis using anti-FlaB antibody generated in mice using complete Freund’s adjuvant (Sigma, CAS9007-81-2) and HRP-conjugated anti-mouse polyclonal secondary antibody (Dako, P0260).

### TLR5-dependent NF-κB reporter assay

To measure the NF-κB stimulating activity of eFlaB with a broad range of protein concentrations (Figs. 1c and 2c), we used HEK-Blue^TM^ hTLR5 cells (InvivoGen, hκb-htlr-5) with HEK-Blue^TM^ detection (InvivoGen, hb-det2) assay systems following the manufacturer’s protocol. We calculated the EC_50_ by using triplicate OD620 nm values for each concentration of protein. To determine the biological activity of deglycosylated eFlaB variants (Supplementary Fig. 3b), we performed in vitro NF-κB luciferase reporter assay using HEK293T cells. HEK293T cells were maintained in DMEM medium (Gibco^TM^, 11995-065) comprising 10% FBS, 100 units/ml of antibiotics (Gibco^TM^, 15140122) at 37 °C, and 5% CO_2_. At least 3 passages of cells were used for the NF-κB reporter assay. Cells were seeded at 2 × 10^5^/well in 24-well plates (SPL Life Sciences, Korea, 30024) for 24 h and transfected with the reporter plasmid pNFκB-Luc (100 ng/well), p3x Flag-hTLR5 (100 ng/well), and 50 ng/well of pCMV-β-Gal using 5 μl/well of Effectene (Qiagen, Germany, 301427) for overnight. The transfected cells were treated with pFlaB, eFlaB, or mutated eFlaB at a stoichiometric concentration and incubated for 24 h. The media were removed from the cultures and treated cells with cell lysis buffer (Promega, Madison, WI USA, E153A). From cell lysates, luciferase activities were measured by a luminometer (Berthold, Lumat-Plus LB96V).

### Deglycosylation of eFlaB by enzyme treatment

The eFlaB were deglycosylated by Peptide N-glycosidase F (PNGase F) enzyme (Promega, Madison, WI USA, V483A) using a non-denaturing form following the manufacturer protocol. Briefly, PNGase F (10 U/μl) was added to eFlaB (15 μg) and incubated at 37 °C for 2 h.

### N-Glycosylation site prediction

We predicted the N-glycosylation sites of FlaB using NetNGlyc-1.0 (https://services.healthtech.dtu.dk/service.php?NetNGlyc-1.0), a bioinformatic tool that determines the N-glycosylation consensus sequence Asn-Xaa-Ser/Thr.

### Site-directed mutagenesis

Site-directed mutagenesis of *eflaB* was performed following manufacturer protocol (Enzynomics, Inc., Daejeon, Republic of Korea, EZ004M). Briefly, pSectag2B plasmid containing *eflaB* gene was used as a template for PCR reaction with specific primers (Supplementary Table 2) designed to mutate from “AAC” or “AAT” (Asparagine, N) to “GCC” or “GCT” (Alanine, A). The mutated *eflaB* sequences were confirmed by Macrogen Online Sequencing System (http://dna.macrogen.com/kor/).

### Co-immunoprecipitation (Co-IP)

Briefly, we transfected the HEK293T cells with the p3XFlag-hTLR5 plasmid for 24 h. The cells were lysed by using RIPA buffer containing protease inhibitors, following we sonicated and then centrifuged to collect the supernatant. The proteins, pFlaB, eFlaB, eFlaB^N83/101/139A^, and eFlaB^N83/101/139/273/330A^, were mixed with supernatant, rotating at 4 °C overnight (complex I). At the same time, we mixed the Protein A/G plus-agarose (Santa Cruz Biotechnology, sc-2003) with an anti-Flag tag antibody (Abcam, ab1162; 1:40 dilution) and rotated it at 4 °C overnight (complex II). Then, we washed complex II with IP buffer (1% TritonX-100, 25 mM Tris pH 7.5, 10% Glycerol, 150 mM NaCl, 1 mM DTT, 1 mM EDTA, and protease inhibitor) 5 times. Next, we mixed complex I with II and rotated at 4 °C for 6 h. Finally, we washed the complex with IP buffer five times to remove the nonspecific binding proteins. After washing, we added 50 μl of 1x SDS loading buffer and boiled it for 10 min. SDS-PAGE and further western blot were analyzed. We have used two different antibodies, mouse anti-FlaB (1:2,000 dilution), and anti-Myc-HRP (Novex®, 46-0709; 1:2,000 dilution), for Western blot assay to detect pFlaB and eFlaB variants, respectively, because anti-FlaB low detected eFlaB proteins due to glycosylation on eFlaB since eFlaB variants were expressed along with Myc-tag, which was well detected by anti-Myc (Fig. 2d).

### Concanavalin A column for an analysis of N-glycosylated proteins

We checked whether only glycosylated eFlaBs bind to concanavalin A (ConA) by using the ConA Glycoprotein Isolation Kit (Thermo Scientific, USA, 89804) following the manufacturer’s protocol. Briefly, we pipetted the ConA lectin resin slurry into the spin column and centrifuge for 1 min at 1,000 × *g* to rinse the resin three times with binding/washing buffer. Proteins were diluted in binding/washing buffer 200 μg in 500 μl, mixed with resin in the column, and rotated at room temperature for 10 min. The column was then centrifuged for 1 min at 1000 × *g* to remove the unbound protein. The resin in the column was washed with binding/washing buffer several times, and 100 μl of elution buffer was added, then rotated the column for 5 min at room temperature. The column was again centrifuged for 1 min at 1000 × *g* to harvest eluted proteins. Unbound and eluted (ConA-bound) proteins were analyzed by SDS-PAGE.

### Intranasal immunization

Seven-week-old female BALB/c mice (ORIENT, Seongnam-si, Gyeonggi-do, Korea) were host-adapted in our facility for one week before pursuing experiments. Mice were subcutaneously administrated with a blend of Zoletil® 50 (Virbac corporation, Carros, France) and Rompun^TM^ (Elanco Animal Health Korea Co.,Ltd.). After mice were anesthetized, we administered intranasally with PBS, split H1N1 antigen only (sH1N1) (Green Cross, Hwasun, Korea), sH1N1+pFlaB, sH1N1+eFlaB, sH1N1+eFlaB^N83A^, sH1N1+eFlaB^N83/101A^, sH1N1+eFlaB^N83/101/139A^, sH1N1+eFlaB^N83/101/139/273A^, and sH1N1+eFlaB^N83/101/139/273/330A^. The vaccine antigens were diluted in PBS to the final volume of 20 μl/mouse, corresponding to the dosage of sH1N1 0.2 μg/mouse and pFlaB or eFlaB 4 μg/mouse. Mice were immunized three times at 2-week intervals, after 2 weeks of final immunization the sera were taken for assay.

### Enzyme-linked immunosorbent assay (ELISA)

The sH1N1, pFlaB, and eFlaB-specific antibody responses were measured by ELISA using mice serum collected two weeks after the final immunization by retro-orbital bleeding. ELISA plates (Corning Laboratories, 3690) were coated with sH1N1 (4μg/ml), pFlaB, or eFlaB (1 μg/ml) in PBS at 4 °C overnight and blocked with buffer [1 mM of EDTA (JUNSEI, 17385S0401) and 0.5% of BSA (Sigma, A2153-50G) in PBS-T] for one hour at room temperature (RT). The serum was serially diluted in blocking buffer and added as 40 μl/well and incubated at RT for 2 h. The plates were washed 5 times and HRP-conjugated anti-mouse IgG secondary antibody (Southern Biotech, 1036-05; 1:2,000 dilution) was added at RT for 1 h. After washing 5 times, the signal was developed by adding 3,3’5,5’-tetramethylbenzidine (TMB) substrate (BD OptEIA, 555214) and stopped the reaction by adding 1 N H_2_SO_4_. The absorbance was measured at 450 nm with a microplate reader (Molecular Devices Corp., Menlo Park, CA). The antibody titer was computed as the reciprocal Log2 value of the serum dilution which yielded 2-fold higher values of absorbance than the blank well.

### Reporting summary

Further information on research design is available in the Nature Research Reporting Summary linked to this article.

### Supplementary information

Supplementary Information

Reporting Summary

## Data Availability

All data generated during this study are available from the corresponding author upon request.
