# Supplementary material for: Deglycosylation of eukaryotic-expressed flagellin restores adjuvanticity
Source: NPJ Vaccines. 2023 Sep 26;8:139. doi: 10.1038/s41541-023-00738-3 (PMC10522637; doi:10.1038/s41541-023-00738-3)

Supplementary Information

Supplementary Figures

(a)

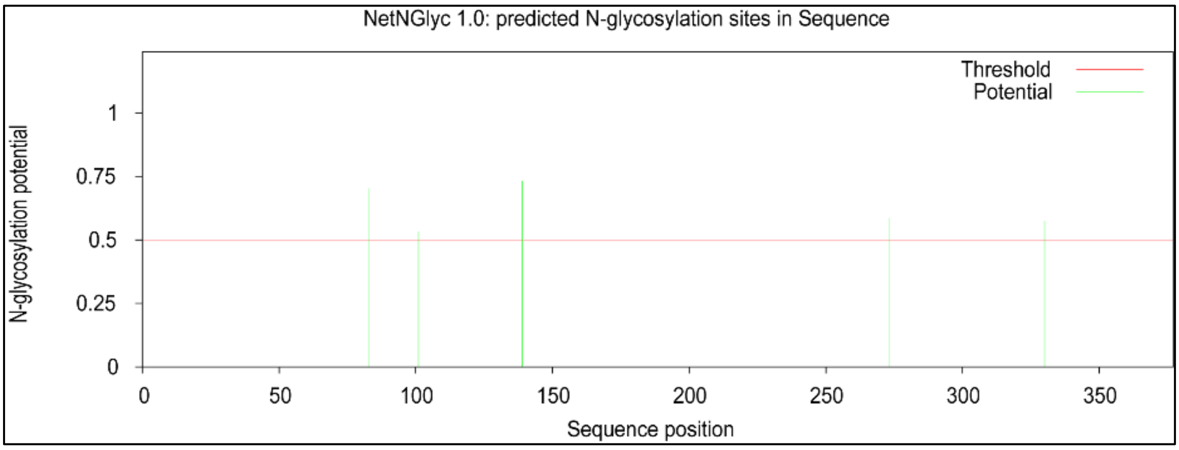

(https://services.healthtech.dtu.dk/service.php?NetNGlyc-1.0)

(b)

```
Name:  FlaB     Length:  377
MAVNVNTNVAAMTAQRYLNNANSAQQTSMERLSSGFKINSAKDDAAGLQISNRLNVQSRGLDVAVRNANDGISIAQTAEG      80
AMNETTNILQRMRDLSLQSANGSNSKSERVAIQEEVTALNDELNRIAETTSFGGNKLLNGTYGTKAMQIGADNGEAVMLS     160
LKDMRSDNVMMGGVSYQAEEGKDKNWNVAAGDNDLTIALTDSFGNEQEIEINAKAGDDIEELATYINGQTDLVKASVGEG     240
GKLQIFAGNNKVQGEIAFSGSLAGELGLGEGKNVTVDTIDVTTVQGAQESVAIVDAALKYVDSHRAELGAFQNRFNHAIS     320
NLDNINENVNASKSRIKDTDFAKETTQLTKTQILSQASSSILAQAKQAPNSALSLLG
................................................................................      80
..N.................N.....................................N.....................     160
................................................................................     240
................................N...............................................     320
.........N...............................................                            400

(Threshold=0.5)
----------------------------------------------------------------------
SeqName      Position  Potential   Jury     N-Glyc
                                 agreement  result
----------------------------------------------------------------------
FlaB           83 NETT   0.7029     (9/9)    ++
FlaB          101 NGSN   0.5326     (7/9)    +
FlaB          139 NGTY   0.7320     (9/9)    ++
FlaB          273 NVTV   0.5860     (9/9)    ++
FlaB          330 NASK   0.5756     (7/9)    +
----------------------------------------------------------------------
```

TLR5 binding motif

(https://services.healthtech.dtu.dk/service.php?NetNGlyc-1.0)

(c)

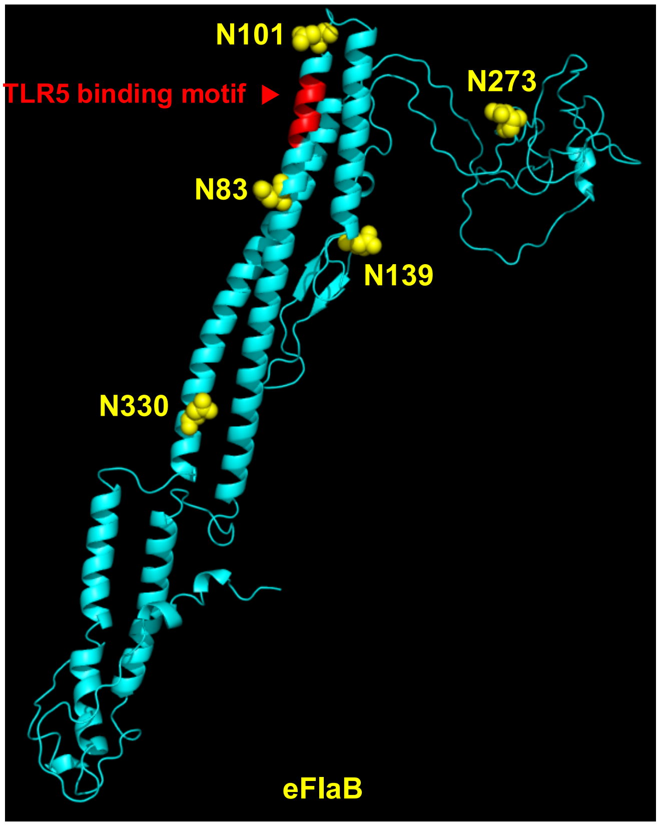

Supplementary Figure 1. N-Glycosylation sites prediction and *in silico* structure analysis of eFlaB. (a, b) The N-glycosylation sites in FlaB amino acid sequence were predicted using NetNGlyc-1.0 (https://services.healthtech.dtu.dk/service.php?NetNGlyc-1.0) online tool. **(c)** Protein structure stimulation of eFlaB by Phyre2 (http://www.sbg.bio.ic.ac.uk/~phyre2/html/page.cgi?id=index) and analyzed by PyMOL2 software.

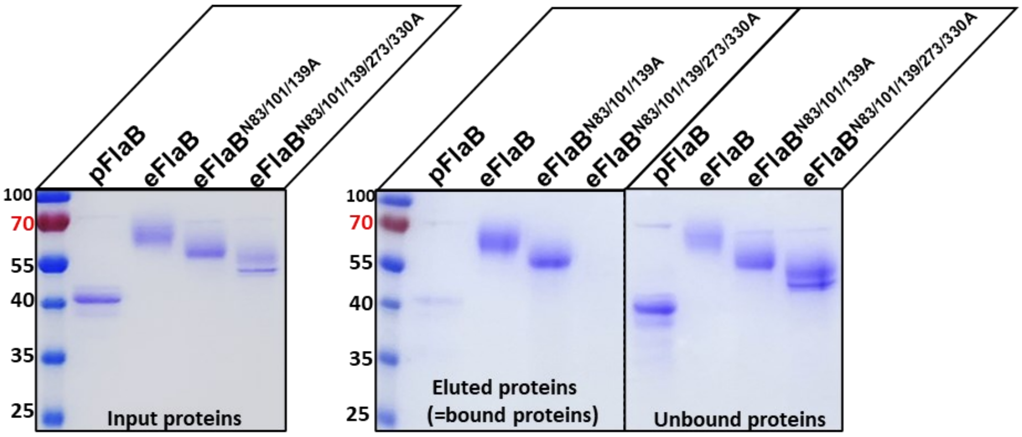

Supplementary Figure 2. The NF-κB activation function of eFlaB and confirmation of N-glycosylation. Concanavalin A column for an analysis of N-glycosylated proteins.

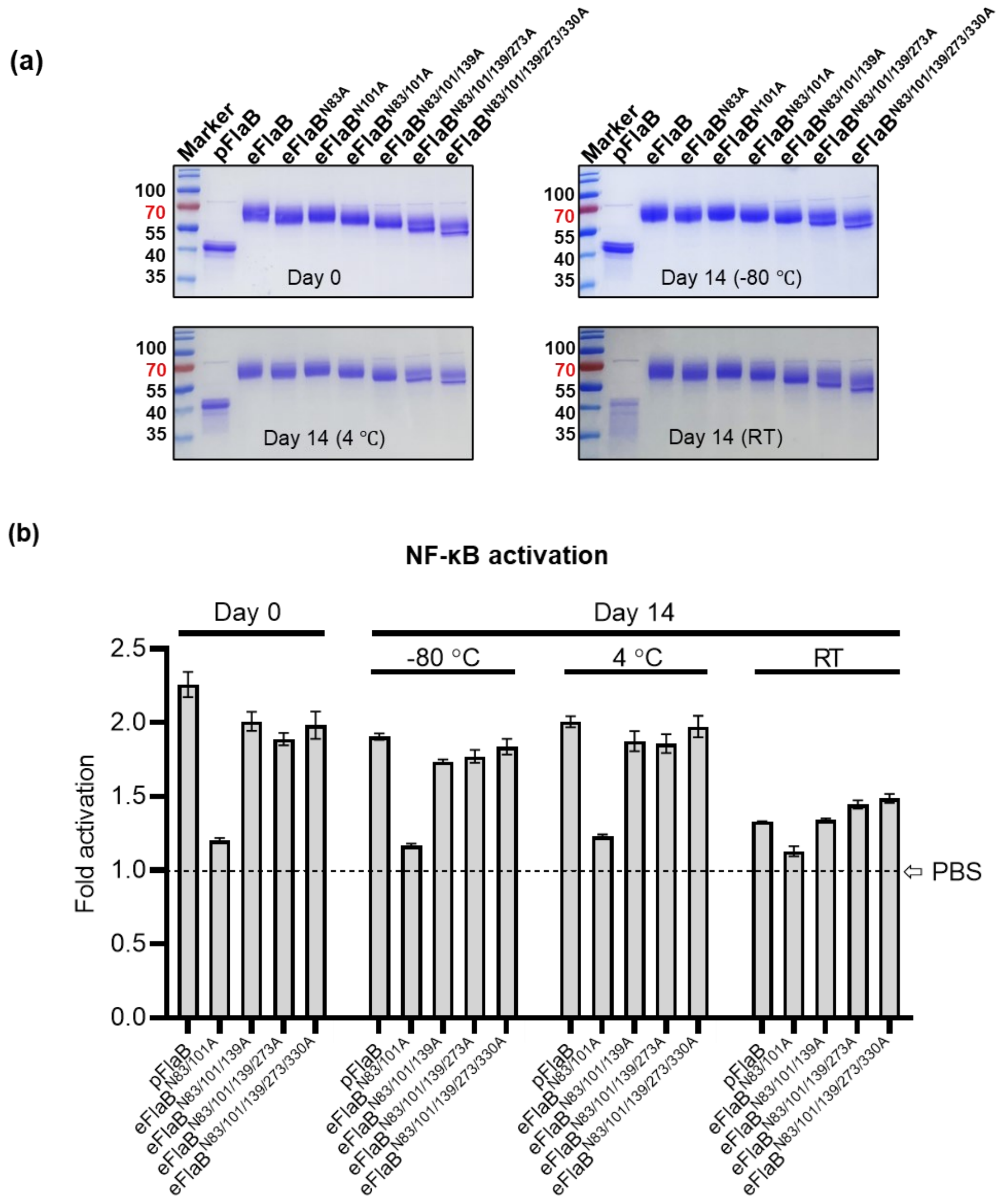

Supplementary Figure 3. Testing the stability of eFlaB. (a, b) pFlaB, eFlaB, and N-glycosylation mutant eFlaB were purified and stored at different temperatures like -80 °C, 4 °C, and room temperature for two weeks. The structural stability and functionality of proteins were tested by sodium dodecyl sulfate-polyacrylamide gel electrophoresis (SDS-PAGE) and NF-κB reporter assay respectively.

Supplementary Tables

Supplementary Table 1. Cell lines, Bacterial Strains and Plasmids used in this study.

Cell/plasmid	Description	Source or reference
**Cell lines**		
Expi293F^{TM}	Transient eukaryotic expression cell system based on high-density suspension culture of Expi293F™ Cells in Expi293™ expression medium.	Thermo Fisher Scientific Inc.
HEK293T	The human embryonic kidney (HEK) 293 cell line expresses a temperature-sensitive allele of the SV40 T antigen.	Thermo Fisher Scientific Inc.
E. coli		
ER2566	B F^{-} λ^{-} *fhuA2* [*lon*] *ompT lacZ*::T7*.1 gal sulA11* Δ(*mcrC-mrr*)*114*::IS*10* R(*mcr 73*::miniTn*10*)(Tet^{S})2 R(*zgb-210*::Tn*10*)(Tet^{S}) *endA1* [*dcm*]	New England Biolabs, Inc.
TOP10	F- *mcr*A Δ(*mrr-hsd*RMS-*mcr*BC) φ80*lac*ZΔM15 Δ*lac*X74 *rec*A1 *ara*D139 Δ(*ara-leu*)7697 *gal*U *gal*K *rps*L (Str^{R}) *end*A1 *nup*G	Invitrogen
**Plasmids**		
pSectag2B	Eukaryotic expression vector for the secretion of C-terminally 6xHis-tagged proteins; Amp^{r}	Invitrogen
pTYB12	Prokaryotic expression vector used in IMPACT^{TM} protein purification system; Amp^{r}	New England Biolabs, Inc.
pCMM250	pTYB12 vector carrying a 1.5-kb *EcoRI-PstI* fragment of *Vibrio vulnificus flaB* sequence	[16]
pSectag2B::FlaB	pSectag2B vector carrying a 1.13-kb *HindIII-NotI* fragment of codon-optimized wild-type *flaB* sequence	This study
pSectag2B::FlaB^{N83A}	pSectag2B vector carrying a 1.13-kb *HindIII-NotI* fragment of the codon-optimized site-directed mutant *flaB* (N83A)	This study
pSectag2B::FlaB^{N101A}	pSectag2B vector carrying a 1.13-kb *HindIII-NotI* fragment of the codon-optimized site-directed mutant *flaB* (N101A)	This study
pSectag2B::FlaB^{N139A}	pSectag2B vector carrying a 1.13-kb *HindIII-NotI* fragment of the codon-optimized site-directed mutant *flaB* (N139A)	This study

Cell/plasmid	Description	Source or reference
pSectag2B::FlaB^{N273A}	pSectag2B vector carrying a 1.13-kb *HindIII-NotI* fragment of the codon-optimized site-directed mutant *flaB* (N273A)	This study
pSectag2B::FlaB^{N330A}	pSectag2B vector carrying a 1.13-kb *HindIII-NotI* fragment of the codon-optimized site-directed mutant *flaB* (N330A)	This study
pSectag2B::FlaB$^{N83/101A}$	pSectag2B vector carrying a 1.13-kb *HindIII-NotI* fragment of the codon-optimized site-directed mutant *flaB* (N83A and N101A)	This study
pSectag2B::FlaB$^{N139/273/330A}$	pSectag2B vector carrying a 1.13-kb *HindIII-NotI* fragment of the codon-optimized site-directed mutant *flaB* (N139A, N273A and N330A)	This study
pSectag2B::FlaB$^{N83/101/139A}$	pSectag2B vector carrying a 1.13-kb *HindIII-NotI* fragment of the codon-optimized site-directed mutant *flaB* (N83A, N101A and N139A)	This study
pSectag2B::FlaB$^{N83/101/139/273A}$	pSectag2B vector carrying a 1.13-kb *HindIII-NotI* fragment of the codon-optimized site-directed mutant *flaB* (N83A, N101A, N139A and N273A)	This study
pSectag2B::FlaB$^{N83/101/139/273/330A}$	pSectag2B vector carrying a 1.13-kb *HindIII-NotI* fragment of the codon-optimized site-directed mutant *flaB* (N83A, N101A, N139A, N273A and N330A)	This study

Amp^r, ampicillin resistance

Supplementary Table 2: Primers for site-directed mutagenesis

Name	Sequence	Tm (°C)
N83A-F	CTGAAGGCGCCATG**GCC**GAAACCACCAACATC	69.4
N83A-R	GATGTTGGTGGTTTC**GGC**CATGGCGCCTTCAG	69.4
N101A-F	CTCTGCAGTCTGCC**GCC**GGCAGCAACAGCAAG	72.2
N101A-R	CTTGCTGTTGCTGCC**GGC**GGCAGACTGCAGAG	72.2
N139A-F	CAACAAGCTGCTG**GCT**GGCACCTACGGCAC	69.9
N139A-R	GTGCCGTAGGTGCC**AGC**CAGCAGCTTGTTG	69.9
N273A-F	GGGCGAGGGCAAG**GCC**GTGACCGTGGATAC	71.9
N273A-R	GTATCCACGGTCAC**GGC**CTTGCCCTCGCCC	71.9
N330A-F	CAACGAGAACGTC**GCT**GCCTCTAAGTCCAGG	66.8
N330A-R	CCTGGACTTAGAGGC**AGC**GACGTTCTCGTTG	66.8

Unprocessed Figures

Unprocessed Figure 1a, b

(a)

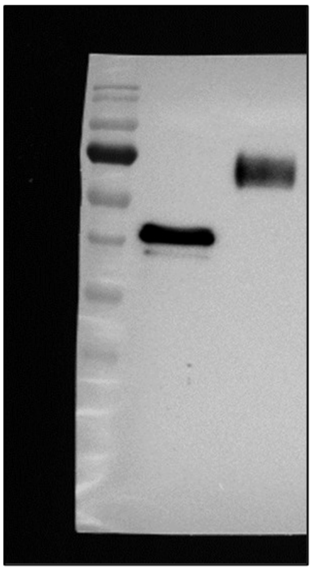

(b)

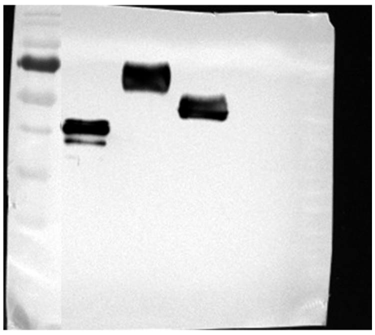

Unprocessed Figure 2b, d

(b)

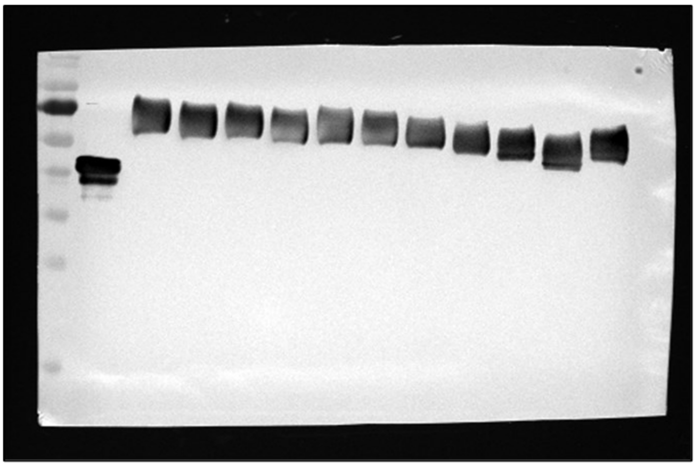

(d)

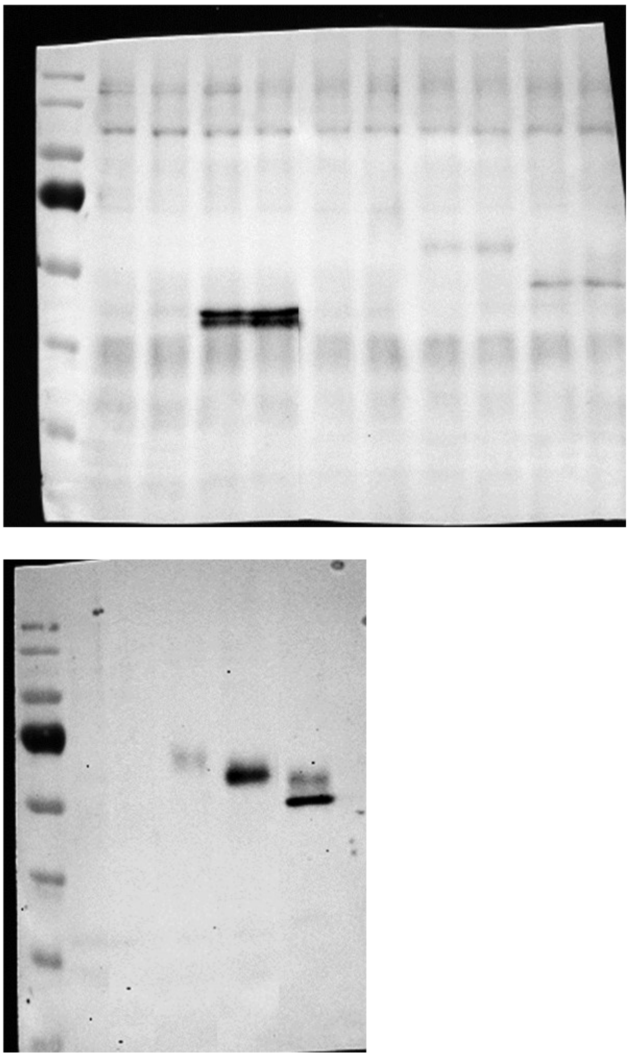

Supplement: Supplementary file 1 — Supplementary Information [file 41541_2023_738_MOESM1_ESM.pdf]
