# Supplementary material for: Deglycosylation of eukaryotic-expressed flagellin restores adjuvanticity
Source: NPJ Vaccines. 2023 Sep 26;8:139. doi: 10.1038/s41541-023-00738-3 (PMC10522637; doi:10.1038/s41541-023-00738-3)
Supplement: Supplementary file 2 — Reporting Summary [file 41541_2023_738_MOESM2_ESM.pdf]

nature portfolio

Corresponding author(s): Joon Haeng Rhee and Shee Eun Lee

Last updated by author(s): Aug 4, 2023

Reporting Summary

Nature Portfolio wishes to improve the reproducibility of the work that we publish. This form provides structure for consistency and transparency in reporting. For further information on Nature Portfolio policies, see our Editorial Policies and the Editorial Policy Checklist.

Statistics

For all statistical analyses, confirm that the following items are present in the figure legend, table legend, main text, or Methods section.

n/a	Confirmed	
☐	☒	The exact sample size (*n*) for each experimental group/condition, given as a discrete number and unit of measurement
☐	☒	A statement on whether measurements were taken from distinct samples or whether the same sample was measured repeatedly
☐	☒	The statistical test(s) used AND whether they are one- or two-sided<br>*Only common tests should be described solely by name; describe more complex techniques in the Methods section.*
☐	☒	A description of all covariates tested
☐	☒	A description of any assumptions or corrections, such as tests of normality and adjustment for multiple comparisons
☐	☒	A full description of the statistical parameters including central tendency (e.g. means) or other basic estimates (e.g. regression coefficient) AND variation (e.g. standard deviation) or associated estimates of uncertainty (e.g. confidence intervals)
☐	☒	For null hypothesis testing, the test statistic (e.g. *F*, *t*, *r*) with confidence intervals, effect sizes, degrees of freedom and *P* value noted<br>*Give P values as exact values whenever suitable.*
☒	☐	For Bayesian analysis, information on the choice of priors and Markov chain Monte Carlo settings
☒	☐	For hierarchical and complex designs, identification of the appropriate level for tests and full reporting of outcomes
☒	☐	Estimates of effect sizes (e.g. Cohen's *d*, Pearson's *r*), indicating how they were calculated

Our web collection on statistics for biologists contains articles on many of the points above.

Software and code

Policy information about availability of computer code

Data collection	1) N-glycosylation sites prediction: NetNGlyc - 1.0 (https://services.healthtech.dtu.dk/service.php?NetNGlyc-1.0)<br>2) Statistical analysis: Graphpad Prism 8<br>3) Western blot analysis: iBrightTM CL1000, Firmware Version: 1.2.5<br>4) Determine NF-kB activation: Spectrophotometer (SpectraMax 190, Molecular Devices Corp., Menlo Park, CA)<br>5) ELISPOT analysis: ImmunoSpot®, Version 6.4.87, Cellular Technology, Shaker Heights, OH.<br>6) ELISA analysis: Spectrophotometer (SpectraMax 190, Molecular Devices Corp., Menlo Park, CA)
Data analysis	All statistical analysis were carried out using the Graphpad Prism 8. iBrightTM CL1000, Firmware Version: 1.2.5 was used for Western blot analysis. The Determination of NF-kB activation was read by a spectrophotometer (SpectraMax 190, Molecular Devices Corp., Menlo Park, CA). HEK-Blue detection was done at OD620. The ImmunoSpot®, Version 6.4.87 (Cellular Technology, Shaker Heights, OH) was used for ELISPOT analysis. The ELISA results were read by a spectrophotometer (SpectraMax 190, Molecular Devices Corp., Menlo Park, CA).

For manuscripts utilizing custom algorithms or software that are central to the research but not yet described in published literature, software must be made available to editors and reviewers. We strongly encourage code deposition in a community repository (e.g. GitHub). See the Nature Portfolio guidelines for submitting code & software for further information.

Data

Policy information about availability of data

All manuscripts must include a data availability statement. This statement should provide the following information, where applicable:

- Accession codes, unique identifiers, or web links for publicly available datasets
- A description of any restrictions on data availability
- For clinical datasets or third party data, please ensure that the statement adheres to our policy

Provide your data availability statement here.

Human research participants

Policy information about studies involving human research participants and Sex and Gender in Research.

Reporting on sex and gender	*Use the terms sex (biological attribute) and gender (shaped by social and cultural circumstances) carefully in order to avoid confusing both terms. Indicate if findings apply to only one sex or gender; describe whether sex and gender were considered in study design whether sex and/or gender was determined based on self-reporting or assigned and methods used. Provide in the source data disaggregated sex and gender data where this information has been collected, and consent has been obtained for sharing of individual-level data; provide overall numbers in this Reporting Summary. Please state if this information has not been collected. Report sex- and gender-based analyses where performed, justify reasons for lack of sex- and gender-based analysis.*
Population characteristics	*Describe the covariate-relevant population characteristics of the human research participants (e.g. age, genotypic information, past and current diagnosis and treatment categories). If you filled out the behavioural & social sciences study design questions and have nothing to add here, write "See above."*
Recruitment	*Describe how participants were recruited. Outline any potential self-selection bias or other biases that may be present and how these are likely to impact results.*
Ethics oversight	*Identify the organization(s) that approved the study protocol.*

Note that full information on the approval of the study protocol must also be provided in the manuscript.

Field-specific reporting

Please select the one below that is the best fit for your research. If you are not sure, read the appropriate sections before making your selection.

☒ Life sciences ☐ Behavioural & social sciences ☐ Ecological, evolutionary & environmental sciences

For a reference copy of the document with all sections, see nature.com/documents/nr-reporting-summary-flat.pdf

Life sciences study design

All studies must disclose on these points even when the disclosure is negative.

Sample size	We performed pilot experiments to determine sufficient sample size for each experiment set.
Data exclusions	No sample or animal was excluded from analyses.
Replication	All experiments were performed at least 3 times reproducibly. The number of samples for each experiment indicated in figure legend. The data shown in the figure panels are the mean of all independent repeated experiments. SDS-PAGE and Western blot picture are from a representative experiment. All the data shown in the manuscript were reproducible in repeated experiments.
Randomization	For in vitro experiment, cells culture were chosen for different treatment randomly and all experiments were performed at least 3 times. For animal experiments, Seven-week-old female Balb/c mice were selected for intranasal immunization.
Blinding	ELISA was performed by individuals (Koemchhoy Khim, Sao Puth) who were blinded to the nature of mice under analysis.

Reporting for specific materials, systems and methods

We require information from authors about some types of materials, experimental systems and methods used in many studies. Here, indicate whether each material, system or method listed is relevant to your study. If you are not sure if a list item applies to your research, read the appropriate section before selecting a response.

Materials & experimental systems

n/a	Involved in the study
☐	☒ Antibodies
☐	☒ Eukaryotic cell lines
☒	☐ Palaeontology and archaeology
☐	☒ Animals and other organisms
☒	☐ Clinical data
☒	☐ Dual use research of concern

Methods

n/a	Involved in the study
☒	☐ ChIP-seq
☒	☐ Flow cytometry
☒	☐ MRI-based neuroimaging

Antibodies

Antibodies used	Western blotting assay<br>Polyclonal Rabbit Anti-Mouse Immunoglobulins/HRP (1:2,000; Dako Denmark A/S; P0260; Lot#20066043)<br>Goat Anti-Mouse IgG(H+L)-HRP (1:1,000; SouthernBiotech; 1036-05; Lot#D4913-XC08D)<br>ELISA assay<br>Goat Anti-Mouse IgG(H+L)-HRP (1:2,000; SouthernBiotech; 1036-05; Lot#D4913-XC08D)<br><br>Co-IP assay<br>Rabbit anti-flag (1:200; Abcam; ab1162; Lot#GR3291118-13)<br>anti-myc-HRP (1:2,000; Novex; 46-0709; Lot#2432522)
Validation	All antibodies used in this study were validated by the suppliers as follows:<br>Poly-isotypes Rabbit Anti-Mouse Immunoglobulins/HRP (1:2,000; Dako Denmark A/S; P0260; Lot#20066043) for WB: species (mouse), manufacturer's website (https://www.agilent.com/search/?Ntt=p0260)<br>Goat Anti-Mouse IgG(H+L)-HRP (1:1,000; SouthernBiotech; 1036-05; Lot#D4913-XC08D) for WB: species (Mouse), application (ELISA), manufacturer's website (https://www.southernbiotech.com/?catno=1036-05&type=Polyclonal#&panel2-1)<br>Rabbit anti-flag (1:200; Abcam; ab1162; Lot#GR3291118-13) for Co-IP, manufacturer's website (https://www.abcam.com/products/primary-antibodies/ddddk-tag-binds-to-flag-tag-sequence-antibody-ab1162.html)<br>anti-myc-HRP (1:2,000; Novex; 46-0709; Lot#2432522) for WB.

Eukaryotic cell lines

Policy information about cell lines and Sex and Gender in Research

Cell line source(s)	Expi293 cells from Thermo Fisher Scientific Inc. (Cat No. A14635)<br>HEK-BlueTM hTLR5 cells from InvivoGen (Cat No. hκb-htlr-5)
Authentication	These cell lines were not authenticated by us.
Mycoplasma contamination	We confirmed that the cell lines were negative for mycoplasma contamination.
Commonly misidentified lines<br>(See ICLAC register)	No commonly misidentified cell line was used.

Animals and other research organisms

Policy information about studies involving animals; ARRIVE guidelines recommended for reporting animal research, and Sex and Gender in Research

Laboratory animals	Seven-week old Balb/c female mice (BALB/cAnNCrlOri) were obtained from the Orient Bio (Orient Bio Co., Republic of Korea).
Wild animals	This study did not use wild animals.
Reporting on sex	Seven-week old Balb/c female mice (BALB/cAnNCrlOri)
Field-collected samples	This study did not use animals collected from the field.
Ethics oversight	All animal experimental procedures were approved by the Chonnam National University Institutional Animal Care and Use Committee under the protocol CNU IACUC-H-2021-51. Animal research facility maintenance and experimental procedures were conducted in rigorous accordance with the guidelines of the Animal Welfare Act legislated by the Korean Ministry of Agriculture, Food and Rural Affairs.

Note that full information on the approval of the study protocol must also be provided in the manuscript.
